# Methods to Make Homogenous Antibody Drug Conjugates

**DOI:** 10.1007/s11095-014-1596-8

**Published:** 2014-12-16

**Authors:** Toni Kline, Alexander R. Steiner, Kalyani Penta, Aaron K. Sato, Trevor J. Hallam, Gang Yin

**Affiliations:** Sutro Biopharma, Inc, 310 Utah Ave Ste 150, South San Francisco, California 94080 USA

**Keywords:** antibody drug conjugates (ADCs), homogenous conjugates, protein engineering, site-specific conjugation, targeted delivery

## Abstract

Antibody drug conjugates (ADCs) have progressed from hypothesis to approved therapeutics in less than 30 years, and the technologies available to modify both the antibodies and the cytotoxic drugs are expanding rapidly. For reasons well reviewed previously, the field is trending strongly toward homogeneous, defined antibody conjugation. In this review we present the antibody and small molecule chemistries that are currently used and being explored to develop specific, homogenous ADCs.

## Introduction

Antibody drug conjugates (ADCs) are a class of biotherapeutics that combine the specificity, favorable pharmacokinetics, and biodistribution of a monoclonal antibody (mAb) with the cytotoxic potency of a small-molecule drug. This combination results in a widened therapeutic window over traditional cancer chemotherapeutics. Thus, many cytotoxic drugs that are not amenable to traditional chemotherapy can often be used in the context of an ADC (1–6).

While the concept of ADCs is in principle a simple one, their practical implementation is dependent on many factors. The conjugation method can be as important as the choice of mAb and cytotoxic drug when designing ADCs, as it potentially impacts efficacy, stability, and toxicity of the resulting product (7, 8).

Given the scarcity of orthogonally-reactive functional groups on natural antibodies, current ADCs on the market are made by conjugating small-molecule cytotoxic drugs to primary amines in lysines or thiols exposed by reduction of interchain disulfide bonds, as is the case for the recently approved ADCs Kadcyla/trastuzumab emtansine/T-DM1 (9) and Adcetris/brentuximab vedotin/SGN-35 respectively (10, 11).

Though these ADCs improve the therapeutic index of the original small-molecule drug and improve the potency over the naked antibody, ADCs made in this manner are heterogeneous mixtures, in which each species has different pharmacological properties. (12). Furthermore, development of safe and efficacious therapeutics requires their accurate characterization throughout all phases of the discovery, optimization, and development life cycle. Since ADCs made by these conventional conjugation chemistries yield heterogeneous mixtures that obscure the individual characteristics of each underlying species, they make optimization to the best possible ADC difficult. For example, much discussion has centered on the optimal number of linker warheads that can be attached to antibodies, without being detrimental to the original physicochemical characteristics. While there will be an approximation of the maximally tolerable payload of specific linkers and warheads, the numbers that have been claimed to be optimal thus far, are based on experimental data with conjugation chemistries utilizing the natural amino acids. Conjugations to available lysines and cysteines are stochastic in number and location, giving a distribution of species with DARs (drug antibody ratios) ranging between 0 and 9 for loading of warhead molecules. For example, Kadcyla, is comprised of the conjugation of an average of 3.4 linker/DM1 warheads per trastuzumab antibody. In the case of huN901-DM1, warheads are conjugated to roughly 40 of the 86 lysines within the trastuzumab antibody sequence corresponding to the accessible sites (13, 6).

The properties of individual species of ADCs in the product can vary depending on the number, position and proximity of the conjugated drugs (7, 8). Cognizant of this, the makers of Kadcyla and Adcetris, and the ADC field as a whole, have migrated toward the development of methods for site-specific attachment of drugs to antibodies, with the intention of further improving ADC therapeutics. The real but still modest increases in the therapeutic index that came about from antibody conjugation may be further increased by defined, designed, and homogenous conjugation of the cytotoxic drugs to the antibody. This review aims to give an overview of the available schemes to generate such homogeneous ADCs.

## Methods to Make Homogenous ADCs

It has been proven that the site of drug conjugation modulates stability, PK and therefore efficacy of ADCs (14). The diverse methods developed to make homogenous ADC can be grouped in two overarching categories. One general strategy requires engineering of the antibody primary structure, *e.g.* mutation of residues to cysteine or a non-natural amino acids (nnAA), or insertion of additional amino acids or fusion tags. The second class uses native antibody sequences and instead employs novel chemistries and linker strategies to yield site-specific modification. The antibody engineering based methods offer the flexibility to mutate or insert tags at multiple defined positions, in order to directly control the DAR and determine the optimal sites for conjugation. On the other hand, methods utilizing native antibodies lack this flexibility and only allow for limited sites to be conjugated, as determined by the chosen chemistry. However, since no mutagenesis of the antibody is required, such methods offer the advantage of slotting directly into existing antibody production platforms, allowing for the rapid and efficient conjugation of any off-the-shelf or *de novo* antibody, without the need for proprietary expression platforms.

### Homogenous ADC Requiring Antibody Engineering

#### nnAA Incorporation

On a conceptual level, the production of homogeneous ADCs with site-specific drug linking requires that the antibody has one or more unique features that can be exploited for attachment of the warhead. One method of incorporating such features in an antibody is the utilization of non-natural amino acids (nnAAs).

##### *In Vivo* nnAA Incorporation

*In vivo* systems that rely on engineered cell-lines have been utilized for incorporating nnAA into antibodies to provide bio-orthogonal conjugation handles. *In vivo* nnAA incorporation relies on a tRNA and synthetase (aaRS) pair that is orthogonal to all the endogenous tRNAs and synthetases in the host cell. The nnAA of choice is supplemented to the media during fermentation, making cell-permeability and stability important considerations for the nnAA. Fundamentally, three approaches based on stop codon suppression have been developed to enable *in vivo* incorporation of nnAAs into antibodies, with amber codon suppression being the most common (15).

One method relies on a tyrosyl aaRS/tRNA pair from *Escherichia coli,* which was engineered to recognize and charge para-acetyl-phenylalanine (pAcPhe). This engineered aaRS/tRNA pair was stably integrated in Chinese hamster ovary cells (16). Subsequent stable integration of light and heavy chain genes containing the amber stop codon was used to express antibodies with pAcPhe at designed sites (17). The keto group of the pAcPhe is reactive towards alkoxy-amines via oxime coupling and can be conjugated with an alkoxy-amine containing linker-drug to generate a site-specific ADC. Ambrx has been an industry leader in *in vivo* ADC production using this approach.

Another approach for *in vivo* nnAA incorporation is based on the natural amber suppressor tRNA/aaRS pair responsible for incorporation of pyrrolysine (Pyl) in *Methanosarcina* species (18). Here, no engineering of the nnRS/tRNA pair is required since the system is naturally occurring. Furthermore, this archea aaRS/tRNA pair is fully orthogonal to both *Escherichia coli* and mammalian cells (19, 20). Serendipitously, the PylRS enzyme is fairly promiscuous in its specificity towards pyrrolysine, and a variety of chemically functionalized pyrrolysine derivatives have been shown to be incorporated by the enzyme (21, 22). Much like the pAcPhe based system, the PylRS/tRNA pair can be utilized to incorporate reactive handles like ketones and azides into antibodies allowing for site-specific conjugation. This approach is currently being developed by Allozyne/MedImmune termed the “AzAbs” (23).

Selenocysteine (Sec) has also been introduced as a chemical handle in antibodies to generate conjugates via transiently transfected human embryonic kidney (HEK) 293 F cells (24). Since Sec is incorporated in naturally occurring systems, no engineering of the aaRS, tRNA or amino acid is necessary to make it suitable for ADC generation. Sec incorporation is accomplished via opal stop codon suppression that is dependent on a selenocystein insertion sequence present on the mRNA transcript. The demonstration of selecocysteine incorporation suggested the potential for site-specific ADCs via selenides.

Site-specific nnAA incorporation can also be achieved by replacing methionine using the sufficiently-promiscuous MetRS. Cells are made auxotrophic for methionine and engineered to overexpress MetRS. Supplementation of methionine analogs into the growth media is used to overcome the 500–2000-fold reduction in catalytic efficiency (K_cat_/K_m_) of MetRS for the nnAAs. Further engineering can be carried out to recover function in the nnAA incorporated proteins (25, 26). So far this particular approach has been exemplified with *E.coli* expression systems, however, the applicability to ADC production has yet to be established.

##### *In Vitro* nnAA Incorporation

The approach of nnAA incorporation into antibodies has also been extended to *in vitro* transcription-translation platforms, although titers and scalability limitations were once a concern for cell-free protein synthesis (CFPS) systems. However, Zawada *et al.* (27) engineered an optimized *E.coli* based cell-free system for predictable high-yield protein synthesis and folding that showed scalability over multiple orders of magnitude. *E. coli* strains and their extracts were engineered to contain all of the necessary components for transcription, translation and energy generation from low cost substrates such as glutamate. Additional improvements upon this CFPS system led to the successful expression of antibodies and antibody fragments in the gram per liter range, in part enabled through overexpression of chaperones and disulfide isomerases in the *E. coli* host strain, from which the cell-free extract is derived (28, 29). In addition, nnAA incorporation strategies used in *in vivo* systems can also be leveraged in *in vitro* cell-free reactions, with the added advantage that that the open nature of CFPS systems allows direct manipulation of the chemical environment in which the protein synthesis occurs. Consequently, cell-free systems can overcome constraints of cell permeability and media stability of the nnAA present in *in vivo* systems.

Another factor common to most nnAA incorporation systems is the presence of competing endogenous release factors (RFs). These RFs naturally recognize certain stop codons and therefore compete for stop codon suppression of nnAA-tRNAs. Thus, full length protein production of nnAA containing antibodies can be significantly reduced in titer when compared to wild-type translation. While removal of the competing RF1 for amber suppression is therefore desirable, this is complicated by the fact that simple deletion of the gene from cells is not tolerated in either microbial or eukaryotic systems. Nonetheless, efficient non-natural amino acid incorporation was achieved by RF1 deletion in *E. coli* enabled by compensatory engineering of RF2 (30). *In vitro* transcription-translation CFPS platforms offer yet another alternative solution, which circumvents the growth rate issues that arise when cell-based systems are subjected to release factor modifications (31). Several groups have demonstrated that RF1 can be removed after extract preparation allowing *E. coli* to have intact RFs during growth of the biomass. Furthermore, RF1 can also be inhibited in the cell-free reaction. These examples enabled improved nnAA incorporation at many sites without reengineering the entire genome to maintain cellular viability (32–36). Hallam and co-workers (37) successfully demonstrated that it is feasible to replace wild-type RF1 with an OmpT-susceptible RF1 in strains of *E.coli*. During the process of extract production, RF1 degrades due to exposure to OmpT, which is localized on the outer membrane and therefore not in contact with RF1 during normal cellular growth. The result is a cell-free system that produces nnAA containing proteins at titers comparable to wild-type, as RF1 function is completely attenuated.

**Fig. 1:**
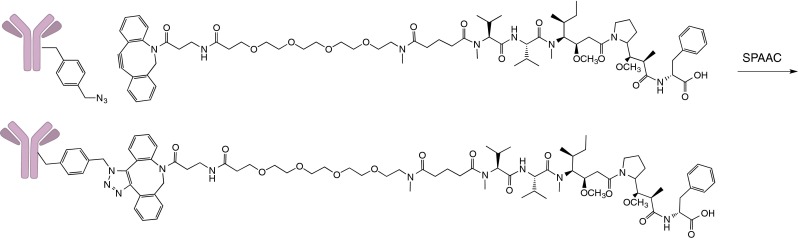
Strain-promoted alkyne-azide coupling (SPAAC) between the inserted non-natural amino acid p-azidomethyl phenylalanine (pAMF) and the dibenzyloctyne (DBCO)-derivitized auristatin drug forms a triazole ring, forging a stable linkage between the small molecule and the antibody.

Thus, CFPS systems can be efficiently leveraged to produce nnAA containing mAbs for ADC generation. Recently Zimmerman *et.al* (38) reported production of site-specific, aglycosylated ADCs by CFPS using a new orthogonal synthetase that enables incorporation of a para-azidomethylphenylalanine (pAMF) followed by strain-promoted alkyl-azide conjugation (SPAAC) to a dibenzylcyclooctyne (DBCO) functionalized monomethyl auristatin F (MMAF) drug (Fig. 1).

To arrive at a synthetase capable of charging pAMF onto its cognate tRNA, the group screened a library of 1760 *Methanococcus jannaschii* tyrosyl- tRNA synthetase (*M.jann*-TyrRS) active site variants, by cell-free expression of each synthetase and subsequent screening for its ability to suppress the amber stop codon in the presence of pAzMeF (38).

Due to the simplified expression format afforded by CFPS systems, rapid screening of many variants can be enabled in microtiter formats. By combining CFPS expression and automated high throughput protein purification techniques, screening of 100’s of site-specifically labeled ADCs was demonstrated with trastuzumab IgG1 as a base scaffold (Sutro Biopharma unpublished data). Top sites within the trastuzumab IgG1 framework were identified based on the attributes of good suppression efficiency, correct assembly of IgG, and high conjugation efficiency while maintaining properties of antigen binding, cell killing, and thermostability. This overall process allowed positional optimization of non-natural amino acids to enable discovery and production of single species ADCs with superior properties and showcases the advantages of homogenous ADC production.

In addition to the Sutro Bipharma technology, PeptiDream has developed a cell free protein expression method. Their Flexizyme system is an *in vitro* evolved artificial ribozyme that catalyzes the aminoacylation of any non-natural amino acid onto any tRNA, thereby functioning as an artificial aminoacyl-tRNA synthetase that efficiently introduces non-natural amino acids into the growing polypeptide sequence during a cell-free translation process (39, 40). This has been integrated with an mRNA display and screening technique called RaPID (Random non-standard Peptide Integrated Discovery), and extensive libraries of peptides have been generated and screened yielding good binding to a variety of protein targets (41). Recently this work was extended to generate a bispecific antibody Fab fragment by introducing *p*-acetylphenylalanine into an αCD3 Fab followed by conjugation to hydroxylamino folic acid. Simultaneous engagement of the T-cell (via CD3) and tumor cell (via the folate receptor) suppressed tumor growth *in vivo* at 5 mg/kg, although regrowth began after 80 days (42). At the time of this writing no ADCs have been reported from this technology.

#### Engineered Cysteine Conjugation

Another option for site-specific modification of antibodies is through the use of cysteines. Since all cysteines in a native antibody are paired in disulfide bonds, free cysteines can be introduced as site-specific chemical moieties through site directed mutagenesis. Due to the highly reactive nature of cysteine as a nucleophile, the free thiols can then be selectively modified through the use of maleimides or other electrophilic groups. Engineering free cysteines into antibodies for site directed conjugation has the advantage that no specialized expression system is needed, as would be the case for nnAA incorporation. However, hurdles for expression and processing have to be overcome. Cysteines are not tolerated at every position in an antibody as they may interfere with correct folding or function, disrupt correct formation of the native disulfide bonds, or form disulfide bonds with free cysteines in other proteins. Another challenge in inserting free cysteines into antibodies is that, even if the antibody is well behaved, the cysteines are often oxidized by disulfide bond formation with small molecule thiols present in the cell, such as free cysteine or glutathione. To liberate the cysteine, the antibody has to be partially reduced, which may result in reduction of some native disulfide bonds. Subsequent re-oxidation must then restore the disulfide bonds to their native configuration, while leaving the engineered cysteine in the reduced form (43). To address the issue of antibody folding, Junutula *et al.* at Genentech developed a Phage ELISA for Selection of Reactive Thiols (PHESELECTOR), which allows for the selection of tolerated cysteine substitutions in the context of an antibody Fab fragment (44). From the PHESELECTOR output, alanine 114 (Kabat numbering) of the heavy chain was determined to be the optimal site for cysteine modification, giving rise to Genentech’s THIOMAB platform. Furthermore, production of various full length antibodies with the A114C HC mutation showed that the cysteine mutation was well tolerated and did not adversely affect antibody function, demonstrating that the approach is applicable across different antibodies. Other sites outside the Fab domain have also been used to create free cysteine containing antibodies (14). Seattle Genetics has an anti-CD33 antibody based on their proprietary engineered cysteine platform in Phase 1 clinical trials, exemplifying the robustness of the approach (45).

#### Enzymatic Conjugation

Ligating enzymes that have high specificity for one substrate but relaxed specificity for the second substrate are ideal for chemoenzymatic synthesis of structurally defined protein conjugates. Bacterial enzymes, many originally identified in highly pathogenic species, have proven extraordinarily useful tools in protein engineering. Mammalian orthologs are either absent or have different substrate specificities. Moreover, genetic manipulation and screening of variants, and expression in non-pathogenic species for *in situ* or *in vitro* applications, is usually straightforward. Mammalian enzymes have also been re-engineered as biosynthetic tools. The chemoenzymatic strategies for protein-small molecule conjugation are presented below in the order of the degree of protein perturbation required, from small (3–6 residues) to medium (12–16 residues) to large (domain fusions).

**Fig. 2:**
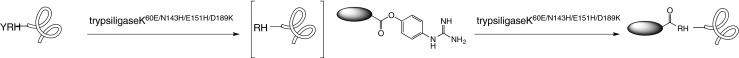
An engineered trypsiligase acylates a newly-created N-terminus at arginine with a small molecule having an activated carboxylate.

A quadruple mutant of trypsin (K60E/N143H/E151H/D189K), trypsiligase, is capable of both Tyr-Arg cleavage and fusion of a ligand *p*-guanidinylphenyl ester (R 4-OGp) onto the newly-exposed arginine amine. Trypsiligase has been shown to efficiently label a variety of proteins modified with an N-terminal YRH tripeptide (Fig. 2). Stoichiometries of the acyl moieties are quite reasonable (3:1), reactions are complete within 1 h, and the engineered hydrolase/ligase accepts a variety of substrates (46).

**Fig. 3:**
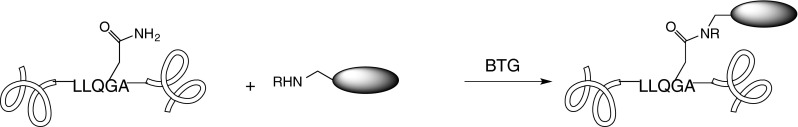
Transglutaminase inserts diverse amines into proteins at suitably exposed glutamines that are embedded in the LLQGA recongition sequence.

*Streptoverticillium mobaraense* transglutaminase, EC 2.3.2.13, is a stable, Ca^+^-independent enzyme catalyzing the γ-acyl transfer of glutamine to the ε -amino group of lysine (47, 48). Chemists at the ETH first observed the selective acylation of amino ligands exclusively by the glutamines located at the flexible regions of an IgG heavy chain. These sites were the mutated (and therefore aglycosylated) N to Q mutation sites, which are highly exposed and available to protein-modifying enzymes. By using the bacterial transglutaminase (BTG) at these sites they synthesized homogeneous radioimmunotoxins that were tumor-uptake selective *in vivo* (49). Building on these results, the Rinat/Pfizer team identified a glutamine tag (Q tag) sequence LLQGA that enabled specific Gln transamination in the presence of all other protein Gln residues and introduced this pentapeptide into discrete locations along an anti-EGFR IgG1 antibody (Fig. 3). Out of 90 sites into which the sequence was introduced, 12 proved to be amendable to conjugation of amine-containing small molecules.

Engineering the Q tag into the anti-M1S1 mAb C16, and subsequent conjugation of monomethyl dolastatin 10 (MMAD) at one heavy chain (HC) and one light chain (LC) site, facilitated comparison of the two discrete DAR 2 ADCs. They observed that in the rat model the LC ADC had more favorable pharmacokinetics. Significantly, they also observed that a higher therapeutic window for both HC and LC ADCs could be achieved *versus* the conventional heterogenous cysteine-linked maleimidocaproyl ValCitpaAB MMAD conjugate. The emphasis in these studies was *site*-dependent rather than *linker chemistry*-dependent stability and the impact of site on overall ADC behavior *in vivo* (50). Subsequently, it was determined that naturally-occurring aglycosylated antibodies gave rise to 1.3% off-target conjugation at the heavy chain Q295 due to conformational effects: in the native glycosylated protein the glycosylation would sterically obstruct this transamination. This exposure effect could be overcome by the Q295N mutation to achieve a completely homogeneous conjugate (51). Thus the Q tag, when presented at solvent-exposed, enzyme-accessible sites, remains a valuable tool for defined homogeneous conjugation. Working with, rather than around, Q295, Innate Pharma recruits bacterial transglutaminase to this site in an aglyclosylated mAb, and introduces the N297S or N297Q mutation to provide homogeneous, site-specific DAR 2 or 4 aglycosylated ADCs (Innate Pharma website).

**Fig. 4:**
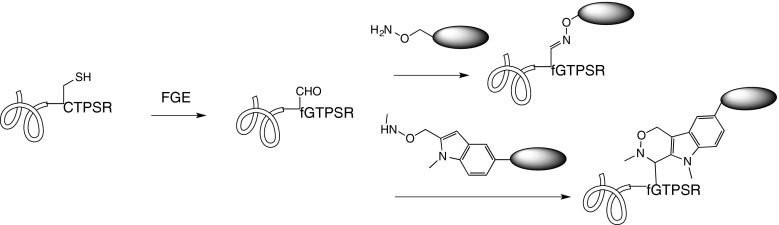
Enzymatic conversion of the cystein thiol to a formyl group enables reactions with oximino and other carbonyl-directed nucleophiles.

The aldehyde tag represents an entirely different chemistry for linking. It is crafted along the same principle of enzymatic recognition of a very small consensus sequences (52). Formylglycine-generating enzyme (FGE) oxidizes the cysteine in the CxPxR sequence at N- or C- termini to formyl glycine, when overexpressed in either mammalian or *E. coli* cells. This enables expression of antibodies with specifically-inserted aldehydes suitable for bioorthogonal reactions with alkoxylamines, hydrazines, or Pictet-Spengler-reacting tryptamines (Fig. 4) (53).

Site specific *in vitro* oximination at an internal IgG CTPSR site has recently been reported, overcoming the requirement that the aldehyde tag be restricted to the protein N- or C-terminus. Interestingly, the recombinant *Mycobacterium tuberculosis* enzyme, used to affect the Cys to fGly conversion in this work, was consumed stoichiometrically rather than reacting catalytically (54). Hydration of the aldehyde in the milieu of protein labeling can lower the efficiency of these reactions (55), but nonetheless Redwood Bioscience/Catalent has successfully developed aldehyde incorporation/reactivity as its SMARTag^TM^ technology, demonstrating its application for a variety of highly controlled protein modifications (56).

Protein modification technologies that have been used for trafficking and biodynamic studies have not to date been used for generation of ADCs, but one may expect that in the future many of these will be adapted for drug bioconjugation. Several of these are included in this review as an acknowledgment of their potential.

**Fig. 5:**
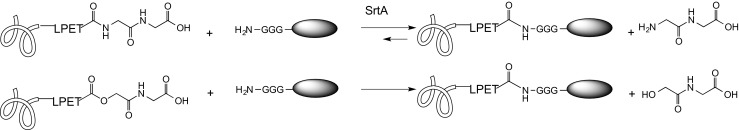
The sortase-mediated glycine splicing places the glycine-derivitized small molecule at the C-terminus of the protein; C-terminal threonine esters show improved reaction kinetcs in this strategy.

**Fig. 6:**
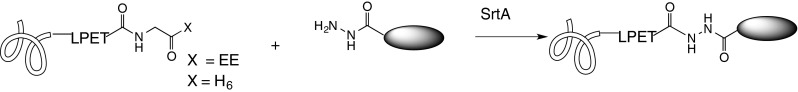
Sortagging using hydrazides in place of polyglycine on the small molecule affords an alternative linkage for the small molecule.

Sortase A (SrtA) from *Staphylococcus aureus*, one of the many related sortases found in a variety of Gram-positive species, is a transpeptidase (57) that recognizes a C-terminal LPXTG sequence, cleaves the TG bond, and facilitates—via the thioacyl enzyme-threonine intermediate—the nucleophilic attack of the incoming protein alpha amine on the threonine. In protein or peptide ligation, the preferred attacking residue is a glycine di- or trimer, and early chemical ligation approaches attached glycines to the small molecule of interest. This was demonstrated to work for conjugation of folate, PEG, rhodamine, cholesterol, biotin, and a variety of peptides, all of which could function with a terminal (Gly)_n_ (58, 59). Eliminating the requirement for oligoglycine, Matsumoto and coworkers (60) used purified *Lactobacillus plantarum* sortase (SrtLp) to site-specifically append the primary amine biotinyl-3,6-dioxaoctanediamine to LPQTx sequences that were not substrates for SrtA. There are some limitations to ‘sortagging’ in the context of ADCs: the conjugation site is restricted to the C-terminus, reaction kinetics are slow, yields are better with glycine than with general primary amines and intramolecular lysines can react to crosslink and aggregate the protein. It has also been shown that the glycine leaving group can return as a competing nucleophile, essentially reversing the reaction, unless a large (*ca* 10:1) stoichiometric excess of intended nucleophile is used. (61). Interest in this approach, however, has prompted creative solutions to many of these impediments, including the use of threonine esters that are recognized as substrates and release the far less nucleophilic glycolic acid, enabling conjugation with a 1:1 stoichiometry (Fig. 5) (62). Directed evolution of the enzyme using yeast-displayed sortase mutants resulted in a 140-fold increase in LPETG-conjugation efficiency (63). Moreover, the potential of protein-small molecule conjugation using sortase was demonstrated by the conjugation of an antibody Fab fragment F19 having a C-terminal LPETGG in the heavy chain to a (Gly)_3_-DY647 fluorophore (64). A recent adaptation of sortase demonstrated the irreversible hydrazinolysis of proteins, enabling the fusion of both protein and small molecule hydrazides at the nascent C-terminus (Fig. 6).

Sortagging has been transitioned from a tool for protein study to a tool for conjugation: with the addition of the second recognition motif of SrtB, NPQTN (65) a dual warhead ADC was designed by the NBE Therapeutics Group. Each heavy chain is C-terminally modified with one antitumor toxin while each light chain is C-terminally modified with a second toxin. Examples of pentaglycine maytansine and pentaglycin auristatins were presented at the 2014 PEGS. The use of cleavable linkers would in principle allow the (Gly)_n_ to be universally acceptable in the linker construction since it would ultimately be separated from the released drug.

**Fig. 7:**
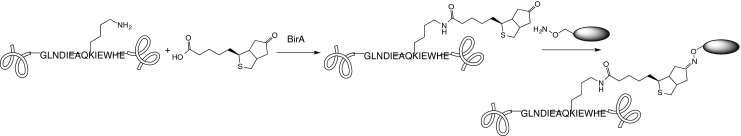
BirA accepts biotin-like ketones for incorporation into the recongition sequence, enabling the carbonyl chemistry that allows oxyamine-small molecules to be inserted into the protein.

The *E. coli* enzyme biotin ligase (BirA), which recognizes the 15-amino acid Avi-tag GLNDIEAQKIEWHE and acylates the embedded lysine with biotin, would at first glance appear useful only in those research applications where site-specific biotinylation was important. The Ting group, however, demonstrated that BirA will accept a synthetic keto analog of biotin as a substrate, albeit at reduced reaction kinetics, and in so doing placed BirA in the same essential category as FGE, *i.e.* an enzyme capable of inserting a carbonyl (in this case a ketone) site-specifically into a protein (Fig. 7). Whereas FGE recognizes a small sequence, but prefers N- and C-termini, BirA requires a longer sequence, but is far less restricted in location along the substrate protein. Reaction with the installed carbonyl was demonstrated using fluorescein hydrazide (66).

The pioneering work in the Ting lab on BirA-based ligation (BLINC) proved difficult to apply to neurons, and the group investigated the potential of *E. coli* lipoic acid ligase (LplA, called “LAP”). Mutating this enzyme to LplA^W37A^ to relax the substrate specificity for the lipoic acid component enabled installation of diverse reactive moieties as long they were at the terminal end of an alkyl carboxylic acid (67–70). An acceptor sequence GFEIDKVWYDLDA was optimized to present the embedded lysine for acylation, effectively converting this lysine into a platform for presenting reactive groups including halides, azides, aldehydes (71), and *trans* cyclooctene (72). Currently both BLINC and LAP technologies have been used for visualization and cellular protein studies rather than for therapeutic conjugates.

#### Fusion Tag Technology

**Fig. 8:**
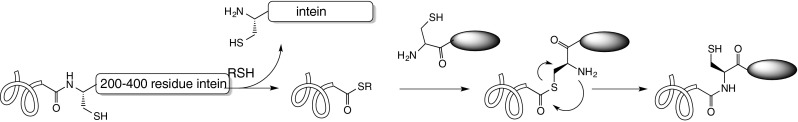
A β-mercaptoamine-derivitized small molecule (typically cysteine-modified) can be appended onto a protein via intein splicing, in which the ultimate bond is a stable amide.

Another strategy for engineering site-specific, homogeneous ADCs involves fusing entire domains or proteins onto the antibody sequence and using their activity to achieve labeling. The most well know such strategy is expressed protein ligation (EPL), which relies on the activity of a self-splicing intein protein to activate the C-terminal of the target protein and therefore allowing formation of a new amide bond with a peptide, protein or small molecule. Though limited to the C-terminus of the expressed protein, the intien is self-excising and leaves only a single cysteine residue scar in the resultant ligated protein. EPL follows a mechanism of (a) C-terminal thioester formation from an intein sequence (*e.g. Mycobacterium xenopi* Gyr A) that spontaneous undergoes N to S-rearrangement, (b) selected thiol displacement of the intein to give an activated thioester, (c) thiol exchange of the thioester with an N-terminal cysteinyl or β-amino mercapto ligand (second protein, peptide, small molecule), followed by (d) spontaneous N-to-C rearrangement to an amide bond that forges the covalent link from the C terminus of the protein of interest to the small molecule(or ligand), with one intervening cysteine (Fig. 8).

Protein trans-splicing (PTS) uses an intein variant which is split into two parts, attached to the N- and C- termini of the partners to be ligated. A similar series of reactions attaches the 2 partners, while excising the intein (73). Intein splicing was demonstrated to site-specifically biotinylate the C-terminus of an antibody directed against the tumor antigen fibronectin ED-B9 (73) and to attach a peptide to an αDEC205 antibody (74). A recent modification of intein chemistry replaced the entire intein with an α-hydroxy acid, incorporated using pyrrolysyl tRNA synthetase, that could be synthetically converted to a C-terminal hydrazide, a function more generally stable than a thioester, but still a good leaving group for acylation chemistry (75). Although intein splicing has been used for site-specifically inserting quantum dots into proteins (76) and for synthesizing a selenoprotein (77), it has not been placed in the toolbox of antibody drug conjugation.

**Fig. 9:**
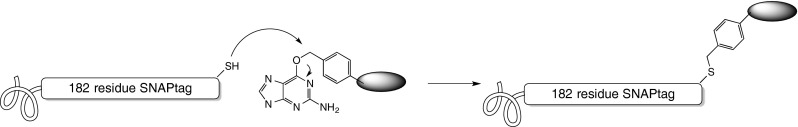
SNAP tagging uses guanine as a leaving group, forging a thioether bond to a benzyl-derivitized small molecule, but requiring a substantial additional polypeptide to be added to the protein undergoing modification.

Proteins that can function with a significant insertion can exploit the O^6^ alkylguanine-DNA-alkyl transferase (AGT) reaction in which the functional group attached to an O^6^ benzyl group is covalently attacked by the cysteine on AGT and thus transferred to the protein-of-interest-AGT fusion (Fig. 9).

To realize the potential of this reaction, directed evolution of human AGT was used to arrive at a mutant enzyme with kinetics comparable to wild type hAGT, while retaining the promiscuous substrate tolerance for the O-benzyl moiety (78). Referred to as the SNAP-tag, this 20 kDa fusion has found wide acceptance for site-specifically modifying proteins for chemical biology studies and therapeutics. An scFv directed against the tumor-associated epidermal growth factor receptor (EGFR) was fused to an AGT SNAP-tag and this fusion protein reacted with a benzyl derivative of the photosensitizer e-chlorin. The scFv-SNAP-e-chlorin conjugate, at a DAR 1, was used in photo-immunotherapy experiments against target and non-target cells and showed strong selectivity, good potency, and dependence on light as well as EGFR expression for cell killing (79).

### Homogenous ADCs from Native Antibodies

All the methods mentioned above to make homogenous ADCs require antibody engineering. More conveniently, other types of methods don’t require protein mutagenesis for site-specific conjugation. The biggest advantage of such methods is to conjugate antibodies at a post-translational stage, which allows the rapid and efficient conjugation of any off-the-shelf or *de novo* antibody.

#### Crosslinking Two Reduced Cysteines

**Fig. 10:**
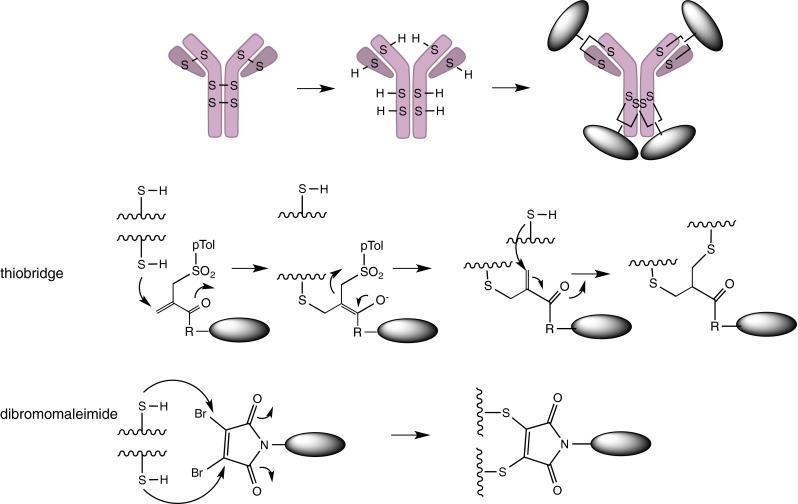
Two solutions to the disulfide re-bridging problem: The thiobridge uses two seuquential reactions and gives rise to a longer, more flexible bridge. The dibromomaleimide could in principle be simultaneously or sequentially attacked by the two thiols, giving rise to a short, rigid bridge.

Instead of using engineered cysteine residues, mAbs can be partially reduced to break intermolecular disulfide bonds of the native cysteine thiols followed by *bis*-alkylation to introduce a three-carbon bridge to which the drug is covalently attached (Fig. 10). Depending on the degree of reduction, the numbers of free cysteines for conjugation can be 4 or 8 to generate DARs of 2 and 4 respectively. PolyTherics demonstrated that the ThioBridge ADCs are more stable than maleimide ADCs in the presence of human serum albumin (80).

Similarly, Igenica uses this approach to reduce all intermolecular disulfide bonds, exposing 8 cysteines, and then uses dibromomaleimide (DBM) to react with the free thiol groups to produce a dithiomaleimide (DTM) (81, 82). Four cytotoxic drugs with this bifunctional linker are conveniently attached to the mAbs by crosslinking the reduced cysteins in the Fab and hinge regions. To make ADCs with DARs higher than 4, additional cysteines must be engineered into the mAb.

#### Glycoengineering

ADCs can also be constructed by attaching the drug to chemically activated sugars, attached to the glycosylated mAb. These glyco-ADCs are constructed by engineering glycosyltranserases in order to accommodate chemically active sugars. Molecules of choice can then be conjugated to the chemical handle on the sugar moiety. Residues in the active site of the glycotransferase are mutated to allow the transfer of the chemically reactive sugar residue.

**Fig. 11:**
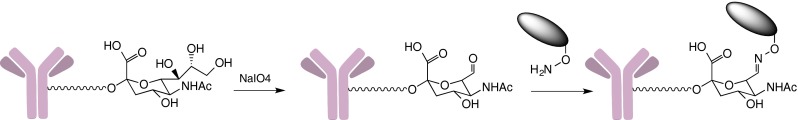
Periodate oxidation of antibody glycans is another strategy to enable carbonyl–directed chemistry.

Using modified galactosyl- and sialyltransferases *in vitro*, terminal sialic acids were introduced into native glycans on Asn-297 of antibodies. Subsequent periodate oxidation then exposed aldehyde groups, which were used to conjugate aminooxy functionalized cytotoxic agents via oxime ligation (Fig. 11) (83). Direct introduction of a keto group was realized by NCI researchers using a mutant β1,4Gal-T1-Y289L transferase, 2-(2-oxopropyl) galactose, and a bioincorporation strategy combined with oximination of the drug-linker (84). SynAffix reports that their GlycoConnect^TM^ technology involves two distinct steps of (a) enzymatic glycan remodeling (‘tagging’) and (b) selective chemical conjugation. The enzymatic incorporation of different sugars enables different conjugation methods: such as azide-containing sugars, thiol-modified sugars and halogenated sugars (SynAffix website).

#### Enzymatic Conjugation by Transglutaminase/Engineered Transglutaminase

As discussed in the section 1.3 on enzymatic conjugation, the use of bacterial transglutaminase (BTG) allows one to attach diverse compounds at multiple positions by inserting an LLQGA tag into antibodies. Deglycosylated mAb can be attached with amino containing compounds site-specifically at its Gln295 residue (49). Unfortunately with intact sugar chain, no Gln residues are available to be reactive with the BTG active site. Using rational design, Dophen Biomed engineered a BTG by enlarging the opening of the active site pocket so that a surface Gln could be docked close to catalytic Cys residue of the engineered BTGenzyme. This enables the direct site-specific conjugation of drug to an un-modified mAb’s heavy chain constant region at a surface Gln residue (85).

We summarize current methods to make homogenous ADCs and the highlights of their technologies in Table 1.

**Table 1:** Regio- and Stoichiometry- Controlled ADCs have been Obtained by a Variety of Methods, Reflecting the Expanded Interest in the Field. KEY: (**a**) Whether the Antibody is (yes) or is not (no) Glycosylated. (**b**) The Expected Drug-Antibody Ratio Based on Available Sites on the Antibody. In Many Cases, the DAR may be Somewhat More Variable than the Average Values Presented Here

Mutagenesis	Method and Conjugation chemistry		Glycosylation^a^	Conjugation site	DAR^b^	Institutions
Yes	Mutation introduced					
	Engineered Cys	Thioether via thiol-maleimide	Yes	Engineered Cys	2 or 4	Genentech, Seattle Genetics
	Enzymatic manipulation	Transamination onto inserted glutamine	Yes	LLQGA	2 or 4	Rinat/Pfizer
		Transamination onto native Q295 (requires N297S mutation or deglycosylation) or N297Q	No	Q295 or Q297	2 or 4	Innate Pharma
		FGE oxidation to aldehyde (SMART tag) by insertion of FGE recognition tag cxpxr to generate formylglycine (Fg) from cysteine; several available carbonyl chemistries	Yes	FgxPxR	2, 4, or 6	Redwood Bioscience/Catalent
	nnAA incorporation	*In vivo* nnAA incorporation via Met or amber suppression aldehyde-hydroxyamine reaction to oxime	Yes	nnAA	2 or 4	Ambrx, Allozyne/MedImmune
		*In vitro* nnAA incorporation [3 + 2]SPAAC and/or inverse electron demand Diels-Alder	No	nnAA	2 or more	Sutro Biopharma
No	Thiol linkage	Tandem thiol reaction on *bis*-electrophile	Yes	Cys	2 or more	Igenica, PolyTherics
	Glycan enzymatic conjugation	Remodel the conserved glycan to recruit carbohydrate functional groups for conjugation to mAb; several chemistries available	Yes	Oxidized glycan carbonyl or synthetic carbohydrate functional group	2	Glykos, SynAffix
	BTG conjugation	Reaction between carboxamide group of glutamine and a primary amino group	No or yes with engineered BTG	Q295	2	Dophen Biomed

### Conjugation Chemistries on the Horizon

**Fig. 12:**
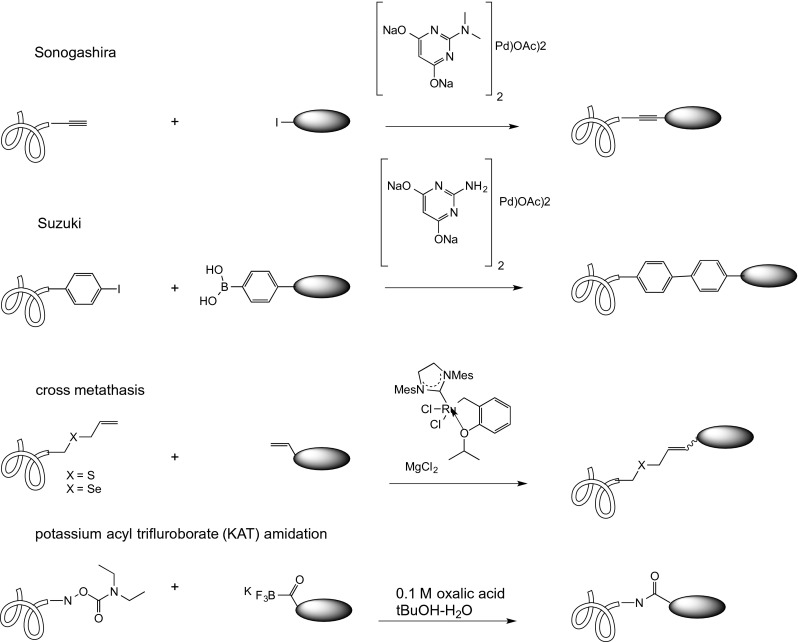
Reactions once considered restricted to synthetic chemistry are being incorporated into the toolkit of bioconjugation. Solubility, stability, and stoichiometry issues are among the parameters that need to be optimized.

Biocompatible synthetic reactions are being explored to extend the toolkit for protein modification. The [3 + 2] strain-promoted azide-alkyne cycloaddition (SPAAC) shown in Fig. 1 was used by University of Georgia researchers in conjunction with glycoengineering. The azide was incorporated into the protein on an azido sugar by a mutated galactosyl transferase. The subsequent reaction with DBCO ligands, including doxorubicin, followed the same course as the Fig. 1 conjugation chemistry (86). Although none of the chemistries described below (Fig. 12) have been applied to ADCs as of the writing of this review, one may look to them for future adaptation to antibody conjugation.

Sonogashira cross coupling of a terminal alkyne to an aryl iodide is in principle a promising bioorthogonal chemistry for ADCs, since either an iodoPhe or an alkynyl aminoacid could be used as the site of conjugation. Li et al. (87) took the latter route, using a homopropargyl glycine auxotrophically substituted for methionine at the C-terminus of ubiquitin. The HPG-Ub protein was converted in 30 min to a variety of aryl-substituted derivatives, in 55–93% yields, in the presence of a water-soluble (and -compatible) palladium complex developed in their group. The 50:1 stoichiometry of both ‘catalyst’ and aryl iodide to protein, however, indicate that further development of this method is necessary before using it to make cytotoxic drug conjugates on antibodies. A recent and impressive extension of Sonogashira reactions in living bacterial cells, at much lower stoichiometires and with a simple Pd(NO_3_)_2_ promoter, was reported by Li et al. (88), suggesting that these developments are attainable. Unfavorable stoichiometry has so far also impeded Suzuki-Miyaura coupling, which has been utilized for the reaction of iodophenylalanine in maltose binding protein to small molecule aryl boronates (89), from becoming a mainstream method for antibody-drug conjugation.

The facile conversion of thiols to allyl thiols, and the reactivity of cysteinyl allyl thiols in cross metathesis, promoted by water–soluble ruthenium catalysts, opened an alternative route to cysteine conjugation. Initial attempts to modify the *B. lentus* subtilisin protein required 10,000:1 stoichiometry of the small molecule olefin, however, due to a strong tendency toward self-metathesis. (90, 91). Binder and Raines subsequently reduced the stoichiometry to 200:1 (92). Se-allyl selenocyseine (Seac) gave somewhat improved kinetics, but nonetheless required 2000:1 small molecule reactant:protein (93). Given the current interest in thiobridging to obtain homogeneous ADCs that require no genetic manipulation, overcoming the hurdles to metathesis would seem a useful pursuit. Chemists at the ETH have been developing potassium acyl trifluroborates (KATs) for bioorthogonal reactions with suitable non-natural amino acids (94) and have recently demonstrated the formation of amide bonds between a diverse set of KATs and synthetic, but unprotected peptides bearing the O-carbamoyhydroxylamine function. (95). The ability to site-specifically incorporate O-diethylcarbamoyl-hydroxylamine into a protein would enable conjugation of small molecules to antibodies via an amide bond.

The pioneering ADCs from the last two decades have become, in turn, the substrate for further innovation. Creative small-molecule and protein chemistries, often co-mingled, are being applied to develop homogeneous, site-specific conjugates. Analytical and bio-analytical technologies are achieving higher and higher resolution to subtend this drive.

Whether derived from a modify-the-antibody side or the modify-the-linker side, the next generation of ADCs to reach the clinic is likely to have a dramatically distinct profile as compared to the current generation of ADCs in the clinic and on the market.
